# Comparative Evaluation of the Antibacterial Efficacy of Aloe Vera, 3% Sodium Hypochlorite, and 2% Chlorhexidine Gluconate Against Enterococcus faecalis: An In Vitro Study

**DOI:** 10.7759/cureus.3480

**Published:** 2018-10-22

**Authors:** Soujanya Goud, Swathi Aravelli, Savitri Dronamraju, Gayathri Cherukuri, Pradeep Morishetty

**Affiliations:** 1 Conservative Dentistry and Endodontics, Army College of Dental Sciences, Secunderabad, IND; 2 Conservative Dentistry and Endodontics, Mallareddy Dental College for Women, Hyderabad, IND; 3 Conservative Dentistry & Endodontics, Mallareddy Institute of Dental Sciences, Hyderabad, IND; 4 Oral Pathology, Panineeya Institute of Dental Sciences, Hyderabad, IND; 5 Conservative Dentistry & Endodontics, Lenora Institute of Dental Sciences, Rajahmundry, IND

**Keywords:** chlorhexidine, colony forming units, enterococcus faecalis, sodium hypochlorite, spectrophotometer, aloe vera

## Abstract

Aim

To compare the antibacterial efficacy of irrigants (aloe vera, 3% sodium hypochlorite (NaOCl), 2% chlorhexidine (CHX), and saline) against Enterococcus faecalis using the turbidometric analysis and colony count method.

Materials and methods

Eighty freshly extracted, single-rooted, human mandibular premolar teeth were taken. Access opening was done, instrumented, and autoclaved. Samples were inoculated with 10 µL of an Enterococcus faecalis (E. faecalis) bacterial suspension and incubated at 37^◦^C for three days. Samples were divided into four groups of 20 teeth each based on the type of irrigating solution used. Group 1 was irrigated with 3% sodium hypochlorite; Group 2 with 2% chlorhexidine; group 3 with aloe vera; and Group 4 with 0.9% saline (the control group). Ten teeth from each group were subjected to a turbidity analysis by spectrophotometer and the remaining 10 teeth from each group were tested for colony-forming units (CFU)/mL. The plates were incubated at 37^◦^C for 24 hours and CFU that were grown were counted using a bacterial colony counter. Results were subjected to an analysis of variance (ANOVA) followed by a post hoc Games-Howell test.

Results

All the tested irrigating solutions demonstrated an antibacterial effect against E. faecalis. The greatest antimicrobial effects were observed in samples treated with 2% CHX (p<0.001). No statistically significant difference was found between 3% NaOCl and aloe vera (p > 0.001) against E. faecalis.

Conclusion

Two percent chlorhexidine exhibited good antimicrobial efficacy against E. faecalis. Three percent NaOCl and aloe vera showed a similar antimicrobial efficacy against E. faecalis. Aloe vera can be used as an antibacterial agent in novel drugs for the treatment of bacterial diseases.

## Introduction

Endodontic infections are known to be polymicrobial in nature, with complex bacterial interactions and a preponderance toward anaerobic species [1]. Bacteria and their byproducts play a relevant role in the onset and perpetuation of pulpal and periradicular disease [2].

The elimination of pulp tissue, bacteria, and microbial toxins from an infected root canal system is one of the cornerstones of successful endodontic treatment [3].

Enterococcus faecalis (E. faecalis) is a gram-positive, facultative, anaerobic, coccal bacterium that plays a major role in the etiology of a persistent root canal infection refractory to endodontic treatment [4]. E. faecalis can survive by genetic polymorphisms and it has got the ability to bind to dentin, invade dentinal tubules, and survive starvation. The inherent antimicrobial resistance and the ability to adapt to a changing environment help E. faecalis to persist in the root canal [5].

Due to the complex anatomy of the root canal system, an effective disinfection in endodontics is only achieved by augmenting mechanical preparation with antimicrobial irrigants. Antibacterial irrigating solutions may reach canal ramifications and inaccessible areas and permeate completely through the dentinal tubules [6].

Sodium hypochlorite (NaOCl) was introduced in endodontics by Walker in 1936. It has been widely used as a root canal irrigant at concentrations ranging from 0.5% to 6%. It is a potent antimicrobial agent effective against bacteria, bacteriophages, spores, yeasts, and viruses. In addition, it effectively dissolves organic debris, inexpensive, has a long shelf life, and is readily available [7].

 Chlorhexidine digluconate (CHX) has been recommended for the root canal irrigation of infected teeth because of its antimicrobial action and substantivity. It is more effective against gram-positive than gram-negative organisms and has proven to be effective in reducing or eliminating E. faecalis from the root canal space and dentinal tubules [8].

Routinely used irrigants like NaOCl and CHX are effective against E. faecalis but they have their own drawbacks like the potential toxicity of NaOCl and the lack of the tissue dissolving property of chlorhexidine [9].

The constant increase in antibiotic-resistant strains and side effects caused by synthetic drugs has prompted researchers to look for herbal alternatives.

Aloe vera is a cactus-like plant that contains 75 potentially active constituents: vitamins, enzymes, minerals, sugars, lignin, saponins, salicylic acids, and amino acids. It has anti-inflammatory, anti-arthritic activity, antibacterial, and hypoglycemic effects [10].

The aim of this in vitro study was to evaluate and compare the antibacterial efficacy of aloe vera with sodium hypochlorite and chlorhexidine against E. faecalis.

## Materials and methods

Eighty, sound, single-rooted, human mandibular premolar teeth extracted for orthodontic treatment were included in this study. The teeth were cleaned using an ultrasonic scaler to render them free from calculus and tissue tags. Conventional access cavity preparation was done using Endo Access diamond point (Dentsply Maillefer, Tulsa, Oklahoma, US). The working length was established 1 mm short of the length where the file exited the apical foramen. Apical preparation was done using K-file (Dentsply Maillefer, Tulsa, Oklahoma, US) up to size 50. Then, step-back preparation was done till size 70. Canals were irrigated copiously with physiologic saline (0.9 w/v sodium chloride) after each instrument. Each canal was then rinsed with 1 ml of 17% ethylenediaminetetraacetic acid (EDTA) (Ammdent, Mohali, India) for one minute followed by 3 mL of 3% NaOCl (Vishal Dentocare Pvt Ltd, Ahmedabad, Gujarat, India) to facilitate the removal of the smear layer. Two coats of nail varnish were applied to the external surface of all roots to prevent bacterial microleakage through the lateral canals. The specimens were autoclaved for 15 minutes at 121˚C.

All the specimens were placed in an Eppendorf tube containing brain heart infusion (BHI) agar, then 10 µL of bacterial suspension ATCC 29212 (American Type Culture Collection) (Himedia Laboratories, Mumbai, India) (1.5x10^8^ colony-forming units (CFU)/mL) was deposited into the root canals of all specimens using a micropipette in a Class II vertical laminar airflow cabinet to prevent any airborne contamination; 200 µL of BHI broth was placed over it to prevent the roots from drying out. The tubes were then covered and incubated aerobically at 37˚C for three days.

After the last inoculation, the contaminated BHI was removed with a sterile syringe, and the canals were dried with sterile paper points. The specimens were randomly divided into four groups of 20 teeth each. Test solutions used were:

Group 1

Three milliliter of 3% sodium hypochlorite for one minute

Group 2

 Three milliliter of 2% chlorhexidine gluconate for one minute

Group 3

Three milliliter of 100% aloe vera for one minute

Group 4

Three milliliter of 0.9% saline for one minute (positive control)

 Irrigation was done with the test solutions. All experimental teeth were then flushed with 30 mL sterile saline to prevent the potential carryover of the irrigants.

After the irrigation, 10 teeth from each group were dried using sterile paper points, which were then placed in a test tube containing a sterile BHI broth. The test tubes were incubated for 24 hours at 37˚C. The occurrence of broth turbidity was indicative of bacteria remaining in the root canal. Broth turbidity was analyzed using an ultraviolet (UV) 3200 double beam spectrophotometer (Labindia Instruments Pvt. Ltd., Thane, India). The spectrophotometer was set to a wavelength of 600 nm. A 500 µL blank sample (BHI broth) was placed in a cuvette. The cuvette was inserted into the machine and the dial was adjusted so that the blank sample had a transmittance reading of zero. After the machine had been zeroed with the blank, the cuvette was removed and was replaced with a cuvette containing 500 μL of the bacterial culture. All measurements were recorded. To confirm the presence of E. faecalis in the turbid broth, a sample was taken from these tubes and cultured on BHI agar plates.

Ten teeth from each group were tested for CFU/mL. After irrigation, a sterile 27 gauge needle was used to collect 0.01 mL (10 µL) of the sample from the canal. Using a bacterial inoculation loop, the bacterial suspension was placed on BHI agar plates. All the 40 agar plates were placed in an incubator at 37˚C for 24 hrs. After 24 hrs, colonies of bacteria were counted using the classical bacterial counting technique and they were counted as the number of colony-forming units (CFU). Colony or viable count per milliliter was calculated by multiplying the average number of colonies per countable plate by the reciprocal of the dilution.

The statistical package for the social sciences (SPSS, version 18, IBM Corp., Armonk, NY, US) was used for the statistical analysis. The mean values were compared by a one-way analysis of variance (ANOVA). A post hoc Games-Howell test was employed to identify the significant groups.

## Results

All the tested irrigating solutions demonstrated an antibacterial effect against E. faecalis. The mean optical density values are the least in Group 2, which is statistically significant in comparison with the other groups (Table 1).

**Table 1:** Comparison of mean optical density of various groups N: number of samples; SD: Standard deviation; p-level of significance; Sig: significance

Group	N	Mean	SD	P-value	Post hoc test
3% Sodium hypochlorite	10	.0305000	.00271825	<0.001 Sig	1>2 3>2 4>1,2,3
2% Chlorhexidine	10	.0131000	.01732339		
Aloe vera	10	.0310000	.00473756		
Saline	10	.0400000	.00567646		

**Figure 1:**
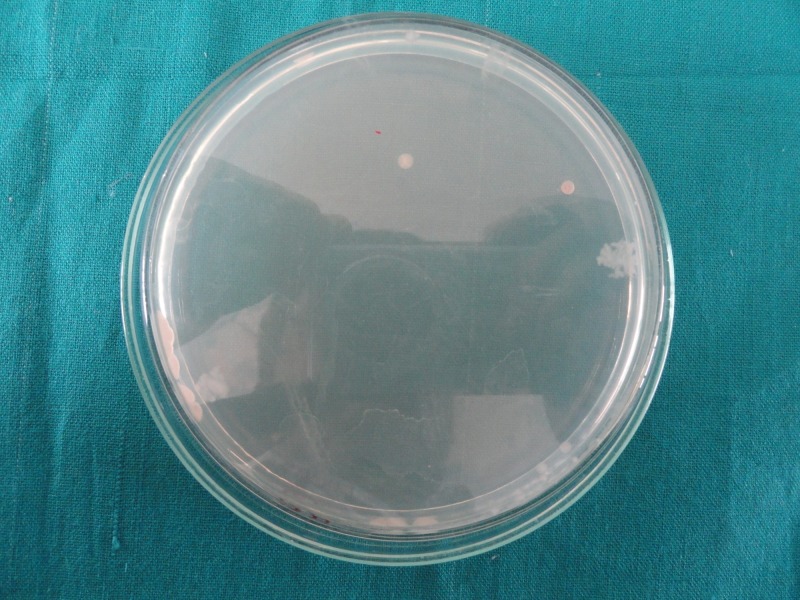
Colony-forming units in Group 2 (2% chlorhexidine)

**Figure 2:**
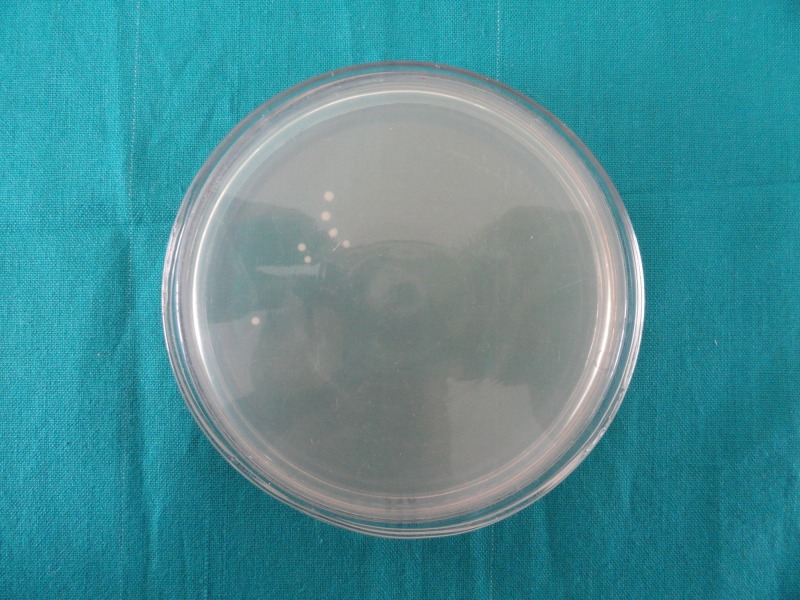
Colony-forming units in Group 1 (3% sodium hypochlorite)

**Figure 3:**
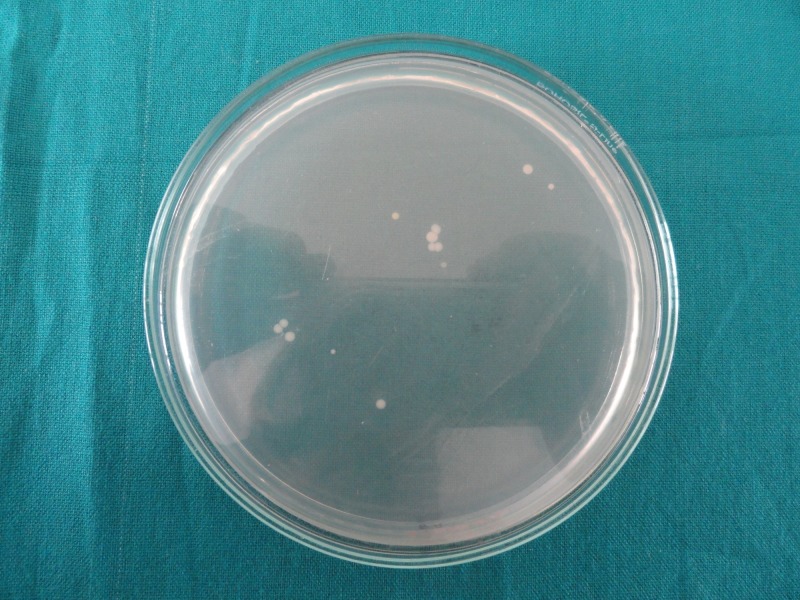
Colony-forming units in Group 3 (aloe vera)

**Figure 4:**
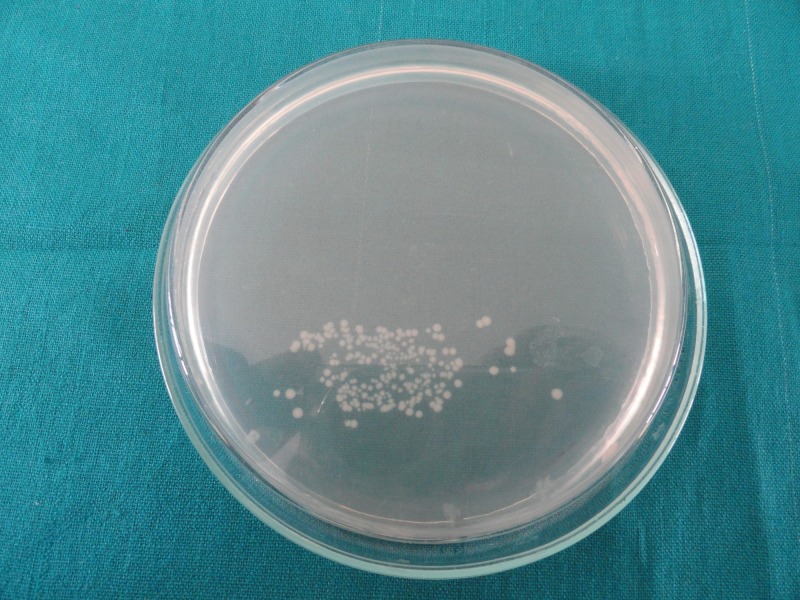
Colony-forming units in Group 4 (saline)

The number of colony-forming units is less in Group 2 (Figure 1) as compared to Group 1 (Figure 2), Group 3 (Figure 3), and Group 4 (Figure 4), indicating good antimicrobial efficacy.

Mean log colony-forming units are less in Group 2, which is statistically significant as compared to Groups 1, 3, and 4. (p<0.001). There was no statistically significant difference found between Groups 1 and 3 (p>0.001) (Table 2).

**Table 2:** Comparison of mean log colony-forming units of various groups N: number of samples; SD: standard deviation; P: level of significance; Sig: significance

Group	N	Mean	SD	P-value	Post hoc test
1. 3% Sodium hypochlorite	10	2.76	0.18	<0.001 Sig	1,3,4>2 4>1,3
2. 2% Chlorhexidine	10	1.36	1.18		
3. Aloe vera	10	2.88	0.09		
4. Saline	10	3.20	0.08		

## Discussion

The prime objective of endodontic therapy is the complete elimination of microorganisms from the root canal system and to create an environment favorable for healing [11].

E. faecalis is the most isolated or detected species from oral infections, including marginal periodontitis, infected root canals, and periradicular abscesses. It is often involved in persistent endodontic infections, has the ability to survive under unusual environmental stress, has been extensively used in experimental work in endodontics, including antiseptic susceptibility, survives in root canals as a single organism without the support of other bacteria, expresses multiple drug resistance, and is easy-to-grow bacteria in the laboratory [12-13].

In the present study, experimental teeth were sterilized in an autoclave for 15 minutes at 121˚C and 15 lb pressure. Machado et al. and Chandra et al employed an autoclave for the sterilization of experimental roots before contamination because bacteria are more susceptible to moist heat, as bacterial protein coagulates rapidly in such an environment [14-15]. The contamination of specimens with E. faecalis, root canal preparation, and sampling procedures on each specimen was carried under a Class II vertical laminar airflow cabinet to prevent airborne bacterial contamination [16]. The in vitro model developed by Orstavik and Haapasalo has been used to assess the antibacterial efficacy of irrigants [17].

The particular concentrations of the 2% CHX and 3% NaOCl experimental irrigants have been used because of their antimicrobial efficacy and minimal tissue toxicity.

One minute of contact time was chosen in this study because less than one minute contact time of the irrigant was not sufficient to eliminate E. faecalis when sodium hypochlorite or chlorhexidine were used [18]. Thirty milliliters of saline was used to eliminate the prolonged contact time of each irrigant and to standardize the groups.

A culture-dependent approach was used, as it is one of the most reliable methods of detecting viable bacteria, particularly when samples are taken immediately after antimicrobial treatment where viability may not be ascertained by most culture-independent methods [19].

The widely used method for the determination of cell number is a turbidometric measurement or light scattering. This technique depends on the fact that as the number of cells in a solution increases, the solution becomes increasingly turbid (cloudy). The solution looks turbid because light passing through it is scattered by the microorganisms present and the turbidity is proportional to the number of microorganisms in the solution. The turbidity of a culture can be measured using a spectrophotometer [20].

In the present study, NaOCl effectively killed E. faecalis. NaOCl exerts its antibacterial effect by inducing the irreversible oxidation of the sulfhydryl groups of essential bacterial enzymes, resulting in disulfide linkages, with the consequent disruption of the metabolic functions of bacterial cells. It also has deleterious effects on bacterial DNA, which involve the formation of chlorinated derivatives of nucleotide bases [19].

Chlorhexidine has good antibacterial efficacy. At low concentrations, CHX has a bacteriostatic effect, at higher concentrations; it has a bactericidal effect owing to the precipitation and/or coagulation of the cytoplasm [6]. The present study results are in accordance with those of Ferraz et al. and Pavlovic et al. who reported that 2% CHX is effective in reducing the bacterial population in infected root canals as compared to NaOCl [21-22].

The aloe vera group has shown a lower number of colony-forming units and turbidity, indicating it had antibacterial activity against E. faecalis, which is statistically similar to NaOCl. Aloe vera has a well-established antimicrobial activity ascribed to compounds that are now specifically identified as p-coumaric acid, ascorbic acid, pyrocatechol, and cinnamic acid [23]. It has antimicrobial activity against Mycobacterium smegmatis, Klebisellapneumoniae, Enterococcus faecalis, Micrococcus luteus, Candida albicans, and Bacillus sphricus [24].

Saline has also shown a considerable reduction in the optical density and CFU counts of E. faecalis. The mechanical shaping and cleaning of the canals and irrigation with saline served to flush out the debris from the canals. This accounted for a reduction in the microbial counts. Kuruvilla and Kamath reported a significant reduction in microbial counts with the usage of saline in canals without mechanical preparation [25].

## Conclusions

Within the limitations of the present study, it can be concluded that 2% chlorhexidine exhibited a good antimicrobial efficacy against E. faecalis. Three percent NaOCl and aloe vera showed a similar antimicrobial efficacy against E. faecalis. Aloe vera can be used as an antibacterial agent in novel drugs for the treatment of bacterial diseases.
